# Psychometric Testing of the Turkish Version of the Stroke Self-Efficacy Questionnaire

**DOI:** 10.1097/jnr.0000000000000308

**Published:** 2019-07-16

**Authors:** Gulcihan ARKAN, Ayse BESER, Meryem OZTURK HANEY, Vesile OZTURK

**Affiliations:** 1 PhD, RN, Lecturer, Faculty of Nursing, Public Health Nursing Department, Dokuz Eylul University, Izmir, Turkey; 2 PhD, RN, Professor, Faculty of Nursing, Public Health Nursing Department, Koc University, Istanbul, Turkey; 3 PhD, RN, Associate Professor, Faculty of Nursing, Public Health Nursing Department, Dokuz Eylul University, Izmir, Turkey; 4 MD, Professor, Faculty of Medicine, Neurology Department, Dokuz Eylul University, Izmir, Turkey.

**Keywords:** stroke, self-efficacy, Rasch analysis, psychometric testing

## Abstract

**Background:**

When stroke survivors return to their lives in society, they often face issues such as physical or cognitive impairment, dependence on others, social isolation, and reduced self-esteem, which may lead to disastrous consequences in patients' self-perceived self-efficacy and self-confidence in everyday life. Self-efficacy plays an important role in the well-being of stroke patients. Accurate assessment of the stroke patients' self-efficacy by health professionals is critical to obtaining data regarding their functioning levels.

**Purpose:**

The aim of this study was to evaluate the psychometric properties of the Turkish version of the Stroke Self-Efficacy Questionnaire (T-SSEQ).

**Methods:**

A sample of 185 stroke patients (mean age = 64.78 ± 10.7) was recruited from a university hospital in Izmir, Turkey. Data were collected between April and October 2016. Translation and back-translation processes were used to translate the T-SSEQ into Turkish. Data were analyzed using the Rasch partial credit model with the Winsteps program to investigate the response scale analysis, tests of fit to the model, unidimensionality, local dependency, item and person separation reliability, separation index, and differential item functioning.

**Results:**

The Rasch analysis showed goodness of fit for both the activity and self-management subscales. Moreover, both scales were identified as being unidimensional in structure. Furthermore, the participants were able to distinguish between the categories of the response options, and scale reliability was supported for both subscales using Rasch analyses.

**Conclusions/Implications for Practice:**

These results indicate that the T-SSEQ is acceptable for use with Turkish stroke patients in both practice and research settings. Furthermore, the questionnaire is suitable for nurses to use in designing interventions and evaluating stroke patients' self-efficacy in clinics, home care, and rehabilitation centers.

## Introduction

Self-efficacy is a predictor of functional dependence, mood, and quality of life after stroke and plays an important role in patients' well-being (Riazi, Aspden, & Jones, 2014). Stroke patients with high self-efficacy are relatively better able to cope with the challenges they face in their daily lives and to adapt to changing living conditions (Maujean & Davis, 2013). In one study of stroke patients, self-efficacy was determined to correlate significantly with participation in activities of daily living, depression, health-related quality of life, walking capacity, stair climbing, and standing up from a seated position. In addition, patients with low self-efficacy have been reported to be more depressed than patients with high self-efficacy. Patients' use of coping strategies and developing a positive outlook have been reported as being related to self-efficacy (Korpershoek, van der Bilj, & Hafsteinsdóttir, 2011). Another study determined that stroke patients with high self-efficacy are highly independent in performing activities of daily living and that the incidence of falls is relatively low among this group (Hellström, Lindmark, Wahlberg, & Fugl-Meyer, 2003).

Stroke patients' awareness of self-efficacy in the rehabilitation process may help them better understand their levels of functioning (Riazi et al., 2014). Therefore, for patients to enhance their self-efficacy, initiatives should be designed to help them learn how to maintain their well-being and to build confidence in their own capacities (Maujean & Davis, 2013). These initiatives have a positive effect on stroke patients' mobility, participation in activities of daily living, depression, and health-related quality of life. Nurses play a particularly important role in these initiatives (Kopershoek et al., 2011). Therefore, accurate assessment and understanding by health professionals, especially nurses, of stroke patients' self-efficacy play an important role in obtaining data about these patients' functioning levels and greatly affect the development of clinical practices and care given at home and rehabilitation centers (Riazi et al., 2014).

A review of the literature revealed that the number of studies investigating the self-efficacy of stroke patients is very limited and that Jones, Partridge, and Reid (2008) developed the “Stroke Self-Efficacy Questionnaire” (SSEQ) for health professionals to measure stroke patients' self-efficacy judgments in the specific domains of functioning. On the other hand, there are no studies or scales in Turkey related to measuring the self-efficacy of stroke patients. Therefore, the aim of this study was to evaluate the psychometric properties of the Turkish version of the SSEQ.

## Methods

### Study Design and Sample

This methodological research was carried out in two stages. First, the English version of the SSEQ was translated into Turkish and was examined in terms of content validity. In the second stage, the psychometric properties of the Turkish version of the SSEQ were evaluated.

The population of the study consisted of outpatients of the stroke clinic of a university hospital in Izmir, a province in the western part of Turkey. The factor analysis that was conducted to evaluate the factor structure of the sample recommends that the size of a study sample be fivefold to 10-fold the number of the items in the scale (DeVellis, 2003; Şencan, 2005; Tavsancil, 2005). Thus, this study should include a minimum of 130 participants, corresponding to 10 times the total number of scale items (13). However, to increase the generalizability of the study, the sample was increased to 185 stroke patients. The sample consisted of individuals over the age of 18 years who were diagnosed with a stroke at least 4 weeks before recruitment and who voluntarily agreed to participate in the study. Those who were not able to read or write Turkish and those not willing to provide/share information with the researchers were excluded.

### Instruments

#### Sociodemographic characteristics questionnaire

This questionnaire consisted of 12 items covering the respondent's age, gender, marital status, educational status, duration since stroke diagnosis, whether receiving poststroke physical rehabilitation therapy, presence of social support, number of people living together, and self-perception of health.

#### Stroke self-efficacy questionnaire

The SSEQ was developed by Jones et al. to measure the self-efficacy judgments of stroke patients related to specific domains of functioning. The scale consists of 13 items that measure the respondent's belief in his or her capabilities. Each item is rated on an 11-point ordinal scale (0 = *not confident at all*, 10 = *very confident*). The range of total possible scores is 0–130. An internal consistency analysis of the scale earned a Cronbach's α of .90. The scale was revised by Riazi et al. (2014) using Rasch analysis. As a result of confirmatory factor analysis, two one-dimensional subscales were obtained, including “activity” (Items 1–8) and “self-management” (Items 9–13). The correlation between the two factors was identified as .58, χ^2^(64) = 247.82, comparative fit index = .82, and standardized root mean square residual = .10, which showed good model fit. The internal construct validity of the scale was examined using Rasch analysis. As a result of this analysis, the item response threshold ordering was considered irregular, so all of the items were recalibrated from an 11-point scale to a 4-point scale. The lowest score in the original scale (0) was kept as the lowest response option (*not confident at all*) in the revised scale, and scores 1–5 were integrated into 1 (*some confidence*), scores 6–9 were integrated into 2 (*moderate confidence*), and the highest score (10) was kept but revalued as 3 (*very confident*). A graphical review of the fit using the item characteristic curve also showed close fit with the Rasch model for all of the items. The *p* values of both subscales for chi-square were insignificant, and the reliability was person separation index (PSI) ≥ .80. Therefore, a good fit was obtained. In addition, neither scale revealed differential item functioning (DIF) in terms of gender or marital status (Riazi et al., 2014).

### Procedures

Data were collected between April and October 2016. Before the study was conducted, approvals were obtained from the Noninterventional Clinical Research Ethics Committee of the university where the study was to be conducted (Approval no. 2016/11-01). In addition, written permission was obtained from the hospital administration of the same university and its stroke outpatient clinic. All of the participants gave their written informed consent. Permission to create and use a Turkish version of the SSEQ was obtained from Professor Fiona Jones by e-mail.

### Data Analysis

Study data were analyzed using SPSS Version 15.0 (SPSS Inc., Chicago, IL, USA) to assess the distribution of the sociodemographic characteristics of the participants in terms of numbers, percentages, and mean values. To determine reliability and validity, the scale was evaluated using Rasch analysis in Winsteps Rasch Measurement Version 3.92.1 (Linacre, 2016). In line with expert opinions, the content validity was assessed using the Davis technique and content validity index (Grant & Davis, 1997). The CVI value calculated based on the opinions of eight experts was 1.00.

### Translation of the Stroke Self-Efficacy Questionnaire and Pilot Study

To ensure the cultural equivalency of the scale, a back-translation method was used. The researchers compared the statements in the original scale with the statements back-translated from Turkish, and those that were adjudged as appropriate were used. After expert opinions were received, the scale was pilot-tested on 10 people and no changes were made in the items. The data obtained from the pilot test were not included in the study (Aksayan & Gozum, 2002; Beaton, Bombardier, Guillemin, & Ferraz, 2007; Erdoğan, Nahcivan, & Esin, 2014).

### Rasch Analysis

Rasch analysis, a modern psychometric method, is a probabilistic mathematical model that estimates item difficulty, person ability, and threshold for each response category on a single continuum logit scale (log-odds units; Hirasawa, Murata, Mayama, & Asaoka, 2014). To perform Rasch analysis, the following criteria should be fulfilled: response scale analysis, tests of fit to the model, DIF, local dependency, reliability, item difficulty and person ability, and a unidimensionality test (Elhan & Atakurt, 2005; Riazi et al., 2014; Tennant & Conaghan, 2007). In addition, if the response category has three or more options, then the Andrich rating scale model or the Masters partial credit model is commonly used in the analysis (Tennant & Conaghan, 2007). In this study, to examine the internal validity of the SSEQ, the following criteria were investigated using the partial credit model, which is one of the Rasch models.

#### Response scale analysis (item response threshold ordering)

Response scale analysis investigates whether response categories have distinct meaning (ordered thresholds in the category probability curves) and whether each category has equal probability to be endorsed by the participants (items evenly spaced). The category threshold is the crossover point between response categories and indicates the point at which the likelihood of choosing either response category is the same (Gadiana & David, 2015). The fit of the responses given to the items with the metric estimates of the underlying structure is determined by whether the threshold values of two consecutive response categories in one item are ordered. If the threshold values are disordered, this shows that individuals have difficulty discriminating between the response categories (Hirasawa et al., 2014; Pallant & Tennant, 2007; Tennant & Conaghan, 2007).

#### Tests of fit to the model

Item fit statistics indicate the degree to which the data fit model expectations. In the WINSTEP program, item fit and person fit are assessed using INFIT and OUTFIT MNSQ (mean square) fit statistics. The quality control limit for MNSQ is between 0.5 and 1.5, which indicates that the scale is unidimensional and that the sample size is adequate. Specifically, an item with a fit statistic > 1.5 means that the item may not contribute to the same underlying construct as the other items in the same scale. An item with a fit statistic < 0.5 means that the item may be redundant in the same scale (Chang, Wang, Tang, Cheng, & Lin, 2014; Lerdal & Kottorp, 2011; Linacre, 2016; Patomella, Tham, & Kottorp, 2006; Tennant & Conaghan, 2007; Wu, Yu, Huang, Hou, & Hsieh, 2016).

#### Differential item functioning

DIF refers to a measurement bias, which is observed when members belonging to different groups with the same latent trait or ability have different probabilities of giving a response on a questionnaire. In other words, DIF is the differentiation in the probability of giving a correct response to one item by individuals in different groups (e.g., gender, socioeconomic level) with similar self-efficacy levels. The Rasch analysis recommends a DIF contrast of between −0.50 and 0.50 logit values with statistical significance and a Mantel–Haenszel chi-square value of *p* ≥ .01 (Linacre, 2016). A negative DIF contrast value indicates that the item is easy for the participants to answer, which will be an advantage for them. In this study, DIF analyses were performed to evaluate the stability of T-SSEQ response patterns between genders (Hagquist, Bruce, & Gustavsson, 2009; Lerdal, Fagermoen, Bonsaksen, Gay, & Kottorp, 2014; Lerdal & Kottorp, 2011; Lin & Pakpour, 2017).

#### Item difficulty and person ability

In a Rasch analysis of a rating scale, difficulty refers to the level of an ability or trait required for agreement with an item. An item with higher difficulty calibrations means a higher level of self-efficacy dimension is required for participants to agree with that item. The Rasch rating scale model reports standardized item difficulties with a mean of 0 and an *SD* of 1 log-odd unit (i.e., logit). A higher logit represents a more difficult item. A negative item difficulty indicates that the associated item is difficult, and a positive item difficulty indicates that the associated item is easy (Chang et al., 2014; Tennant & Conaghan, 2007).

#### Unidimensionality and local dependency

A major assumption of the Rasch model is that each domain is unidimensional (Chang et al., 2014). This means that the answer to an item in the test must be independent of other items. If this assumption cannot be achieved, this affects the unidimensionality of the test and may lead to biased parameter estimates. Moreover, establishing unidimensionality indicates that there is no local dependency. Having local dependency indicates that the response to one item is dependent on a response to another item. There are various ways to test this assumption such as INFIT and OUTFIT MNSQ fit statistics, item point measure correlations, and the principal component analysis (PCA) of residuals. In this study, because the unidimensionality of each of the two subscales is a prerequisite for the conduct of Rasch analysis, a series of PCAs was conducted. The criteria used for dimensionality were that the total variance should be over 50.9 and first contrast in the unexplained variance should be an eigenvalue less than 2.0 in the PCA (Linacre, 2016). Local dependency, which means that some items are still correlated after the same underlying concept has been taken into account, was used in this study and represented by the correlations (*r*) of the Rasch residuals between every two items (Chang et al., 2014). Interitem standardized correlations of ≥ .7 were taken as evidence of high local dependence indicating at least ~50 or more residual common variance between items (Clinton, Alayan, & El-Alti, 2014; Embretson & Reise, 2000; Karim, Shah, Din, Ahmad, & Lubis, 2014; Linacre, 2016; Pallant & Tennant, 2007).

#### Separation and reliability

The person and item separation index statistic value is > 2.0 and > 3.0, respectively (Linacre, 2016). However, a PSI of 1.5 represent an acceptable level of separation (Fisher, 1992). These values indicate whether the scale is sufficiently precise to discriminate between the performances of various individuals. This study applied separation indices to examine how well the PSI could discriminate between respondents and by how well the items could be separated with the item separation index using the scale. In addition, the values for person reliability and item reliability should be greater than .80 and .90, respectively (an acceptable value for person separation reliability is > .70. [Fisher, 1992]). Finally, the Rasch-equivalent Cronbach's α statistic (Kuder–Richardson Formula-20) was evaluated (Linacre, 2016).

## Results

### Characteristics of the Sample

The study sample consisted of 185 participants (37.3% female) with a mean age of 64.78 years (*SD* = 10.7). Nearly half (48.6%) were primary school graduates, 80.5% were living with a spouse and child, 90.8% had a relative providing them with social support, 41.6% had been diagnosed with stroke within the last year, 67.6% were not participating in a physical therapy program, and 55.1% perceived their health as either good or very good (Table 1).

**TABLE 1.**
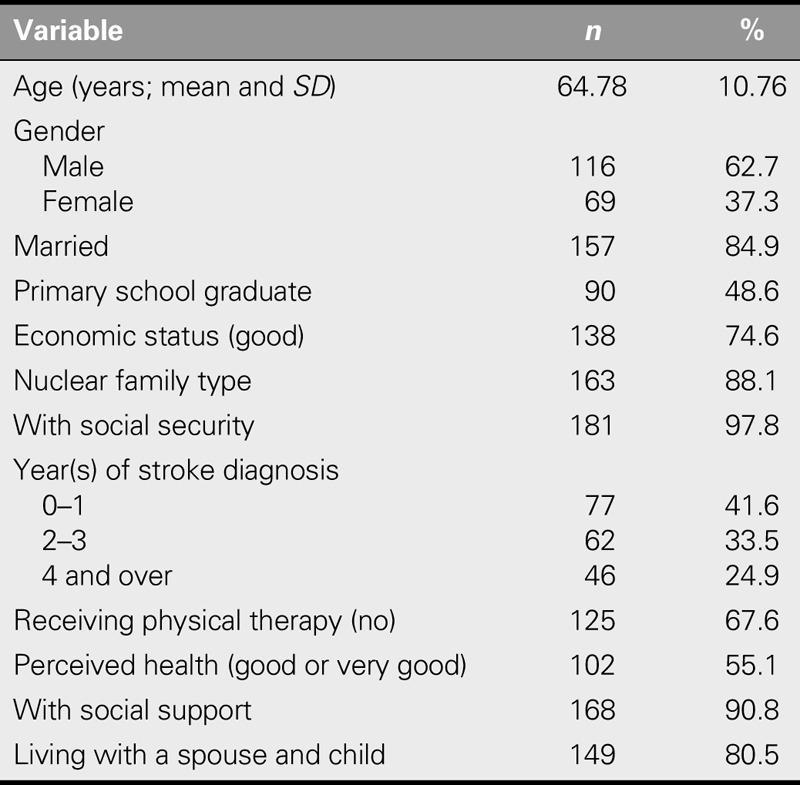
Demographic Characteristics of Participants (*N* = 185)

### Rasch Analysis of the Turkish Version of the Stroke Self-Efficacy Questionnaire

As in the original study, the Rasch analysis of the subscales of the scale was conducted separately within the scope of the internal construct validity studies of the SSEQ. The analysis of both subscales was performed using the unrestricted partial credit model (Riazi et al., 2014).

#### Item response threshold ordering

**Figure 1.**
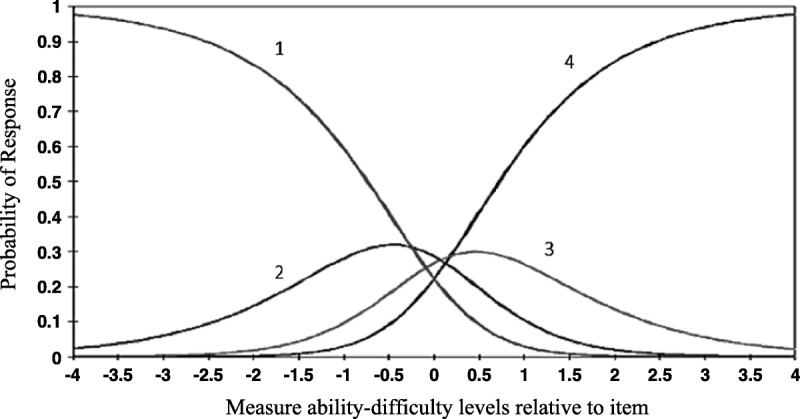
The response category probability curves of the activity subscale. ***Note.*** Item response threshold ordering: 1 = *not confident at all*, 2 = *some confidence*, 3 = *moderate confidence*, and 4 = *very confident*.

**Figure 2.**
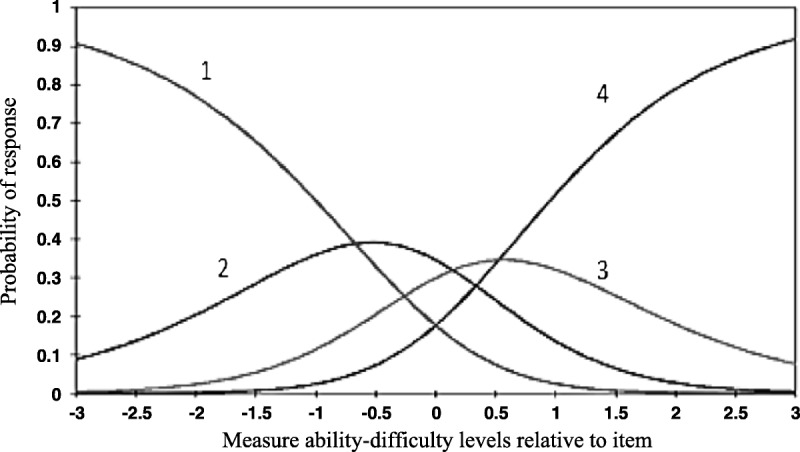
The response category probability curves of the self-management subscale. ***Note.*** Item response threshold ordering: 1 = *not confident at all*, 2 = *some confidence*, 3 = *moderate confidence*, and 4 = *very confident*.

Threshold values were assessed to determine whether the categories of the response options were correctly interpreted by the patients. In both subscales, in transitions from the lowest to highest response categories of the items, it was observed that the threshold values increased as the ability levels increased and that there was nothing unusual (Table 2). As is seen in Figures 1 and 2, on the basis of both subscales, transition threshold values from the lowest response category to the higher response categories increased in parallel with the increases in the participants' stroke self-efficacy levels. In Table 3, the threshold values of all of the items of both subscales are shown separately. These results show that the participants in the study were able to distinguish between the categories of the response options (Table 3).

**TABLE 2.**
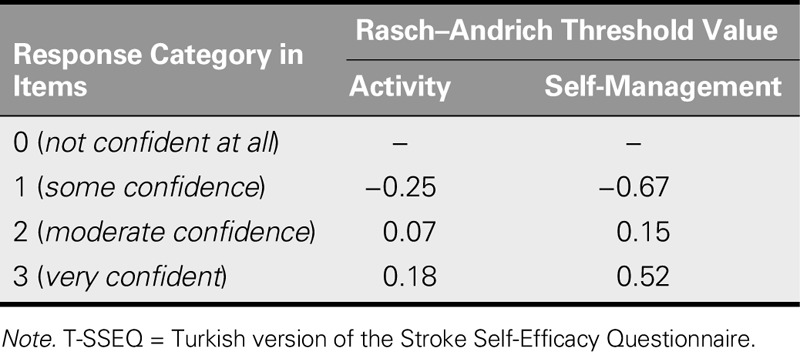
Threshold Estimates for the Subscales of T-SSEQ

**TABLE 3.**
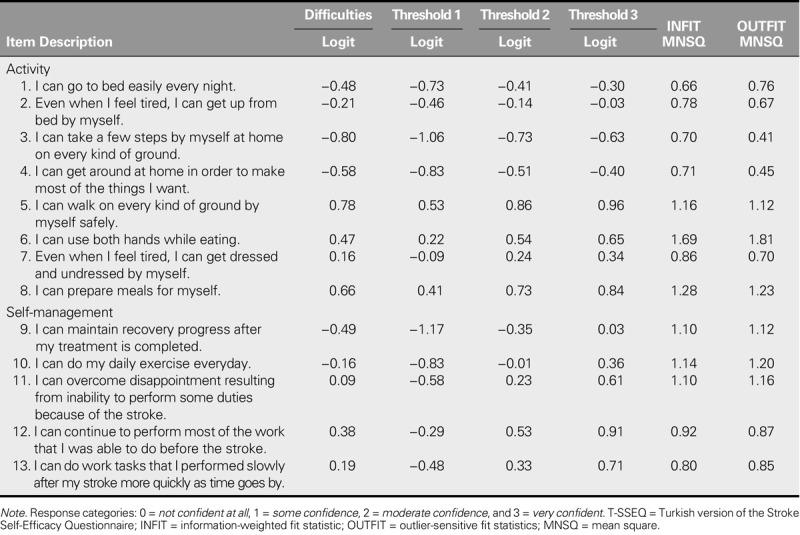
Item Difficulties and Item Fit Statistics for T-SSEQ (*N* = 185)

#### Tests of fit to the model

Goodness of fit for both the activity and self-management subscales to the Rasch model is shown in Table 3. INFIT and OUTFIT MNSQ statistics for all of the items in both subscales showed fit or noise-free calibrations between 0.5 and 1.5. However, Item 6 in the activity subscale had a value that was over 1.5.

#### Differential item functioning

As seen in Table 4, the DIF analysis was performed to determine whether the subscale items were biased by gender. DIF contrast logit values for Items 3, 4, and 8 were higher than 0.50. However, because the Mantel–Haenszel chi-square values (*p* ≥ .01) were not statistically significant, the items in both scales did not show DIF by gender. In other words, the difficulty levels of the items did not differ significantly in terms of gender.

**TABLE 4.**
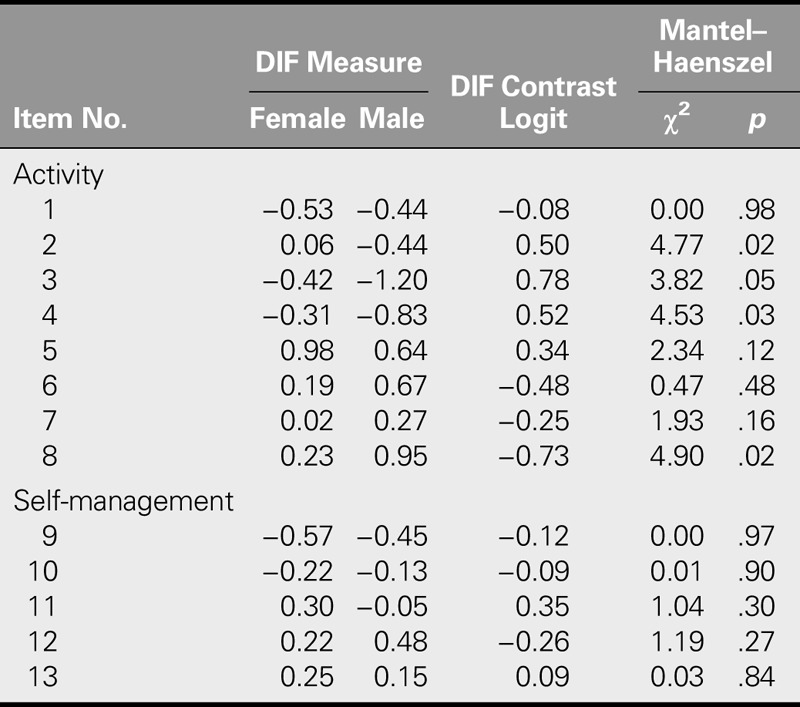
Differential Item Functioning (DIF) Across Genders

#### Item difficulty and person ability

The difficulty levels of the items ranged from −0.80 to 0.78 in the activity subscale and from −0.49 to 0.38 in the self-management subscale. The range of item threshold values was from −0.83 to 0.96 in the activity subscale and from −0.83 to 0.91 in the self-management subscale. No disordering in threshold difficulty was found. In terms of mean item difficulty, Item 5 was the most difficult item and Item 3 was the easiest item in the activity subscale, whereas Item 12 (0.38) was the most difficult item and Item 9 (−0.49) was the easiest item in the self-management subscale (Table 3).

#### Unidimensionality and local dependence

The dimensionality of items in both subscales was examined. In terms of the dimensionality of the eight items in the activity subscale, 53.1% of the raw variance was explained by the measures. The first contrast explained 13.3% of the unexplained variance (first contrast eigenvalue of 2.27). In terms of the dimensionality of the five items in the self-management subscale, 37.4% of the raw variance was explained by the measures. The first contrast explained 18.0% of the unexplained variance (first contrast eigenvalue of 1.14; Table 5). The intercorrelation coefficients of the eight items in the activity subscale varied between .31 and .76, and only four (14%) of the 28 correlation coefficients exceeded .70. On the other hand, the intercorrelation coefficients of the five items in the self-management scale varied between .36 and .66.

**TABLE 5.**
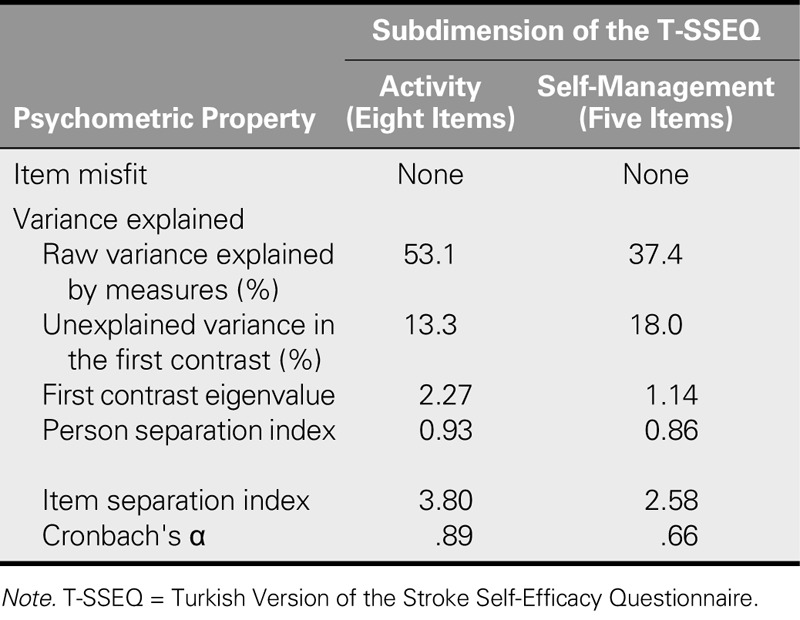
Evaluation of Psychometric Properties of the T-SSEQ Using Rasch Analysis

#### Separation and reliability

Separation and reliability were examined for the two subscales. The PSI was .93 for the activity subscale. The item separation index of 3.80 indicates four levels of item difficulty (“not at all,” “some,” “moderate,” and “very”), and the Cronbach's α value was .89. The PSI was .86 for the self-management subscale. The item separation index of 2.58 indicates four levels of item difficulty (“not at all,” “some,” “moderate,” and “very”), and the Cronbach's α value was .66 (Table 5).

## Discussion

This study evaluated the psychometric properties of the T-SSEQ in a population of poststroke patients. The results indicate that the T-SSEQ has high internal consistency and goodness of fit in Turkish stroke populations.

In this study, the content validity index value for the T-SSEQ was greater than .80, and it was assumed that the expert opinions were consistent with each other and that the items of the scale were appropriate for Turkish culture and measured the relevant issues adequately (Erdoğan et al., 2014; Gozum & Aksayan, 2003; Grant & Davis, 1997; Şencan, 2005).

The criteria to be achieved using Rasch analysis in the internal construct validity of the T-SSEQ were evaluated in detail. The results obtained in this study show that, in the transition from the lowest to highest response categories of the items in both subscales of the 4-point T-SSEQ, increases in ability levels were associated with increases in the threshold values. In other words, the participants were able to discriminate between the response categories. Likewise, for most of the 11-point items of the Rasch-modified SSEQ, the item response threshold ordering was considered irregular. Therefore, the researchers converted the scale items from the original 11-point scoring system over to a 4-point scoring system and found that the response thresholds of all of the items were ordered regularly (Riazi et al., 2014).

Both subscales of the Rasch-modified SSEQ fit the Rasch model well (Riazi et al., 2014). Except for Item 6 in the activity subscale of the T-SSEQ, all of the items in both subscales fit the model well. However, the fit statistics of Item 6 is within reasonable range (Linacre, 2016). These results show that all of the items of the T-SSEQ contribute to successful measurement and that each item score represents the self-efficacy of stroke patients.

The fact that patients with the same level of self-efficacy did not show DIF by gender revealed no gender bias in responding to the items correctly, as all DIF contrasts were less than .5 and nonsignificant. Similarly, no DIF was identified on the Rasch-modified SSEQ (Riazi et al., 2014).

The items in both subscales of the T-SSEQ were found to be generally easy to answer. The most difficult items for the participants were Item 5 in the activity subscale and Item 12 in the self-management subscale. Given that the patients regarded these items as difficult because of the limitations that they faced in performing physical activities due to stroke and because of their reliance on others to perform activities of daily living, it may be assumed that the scale items are well targeted to assess self-efficacy in stroke patients.

The presence of the two unidimensional subscales by Jones et al. (2008) reported that the original scale had a one-factor structure. On the other hand, in the Rasch-modified SSEQ, Riazi et al. (2014) obtained two unidimensional subscales (“activity” and “self-management”) and stated that the model had good fit. In this study, when examining the unidimensionality of items in the activity subscales, as more than 50% of the raw variance was explained by the measures, the presence of a second dimension was not expected. For unidimensionality of the self-management subscale, raw variance explained by measures is poor. However, the first contrast eigenvalue of this subscale is 1.14. It is thought that this may be due to the fact that the subscale has only five items and a second dimension was not expected and was not found. According to these results, it is believed that retests that use larger sample numbers should have larger percentages of variance explained by the measures.

To examine the local dependence of the items, interitem correlations in the activity and self-management subscales were estimated and checked to assess whether correlations fell below *r* ≤ .70 (Clinton et al., 2014; Linacre, 2016). In this study, the interitem correlations for both subscales of the T-SSEQ were less than .70, indicating that no item pairs shared half or more of their random variance. In other words, the low levels of interitem correlation evidenced the assumption of no local dependency in either of the scales and that both scales measure unidimensionalities. Similarly, the local independence of its two subscales proved the unidimensionality of the Rasch-modified SSEQ (Riazi et al., 2014).

The Cronbach's α value of the SSEQ was significantly higher in Jones et al. (α = .90) and the Chinese version developed by Lo, Chang, and Chau (2016; α = .92). The Rasch-modified SSEQ was shown to have a high PSI, and it was furthermore proven that the scale was precise enough to discriminate between the self-efficacy levels of individual patients (Riazi et al., 2014). In this study, for both subscales, the PSI value was less than that required for the "good level" distinction, it did not discriminate between the self-efficacy levels of the patients in the subscales. This was probably due to the small number of items in the subscales. The item separation index indicates whether the sample is large enough to confirm the item difficulty hierarchy of the scale. This study proved that the sample size was sufficient to confirm the item difficulty hierarchies of both subscales. Furthermore, whereas the Cronbach's α for the person raw score test reliability was higher for the activity subscale, it was moderate for the self-management subscale, which was probably due to the low number of items in the latter. These results show that the items of the scale adequately measured the relevant issues, that the items related to the subject, and that the scale was reliable when used on a Turkish sample.

### Implications for Practice

Questionnaires are suitable data-gathering tools for use in outpatient clinic, home care, and rehabilitation center settings by nurses and other health professionals to accurately measure and understand the self-efficacy of stroke patients, obtain data on these patients' functioning levels, and assess self-management initiatives. In addition, the T-SSEQ sheds some light on the use of Rasch analysis in health sciences research.

### Conclusions

The Turkish version of the SSEQ was shown to have constructive psychometric properties and to be a valid tool for assessing the self-efficacy judgments of poststroke patients related to their activities of daily living and self-management abilities.
